# Stimulated UCPCR Levels Are Lower in People With Type 1 Diabetes Than in Other Diabetes Types in Sub-Saharan Africa: Results From a Preliminary Cross-Sectional Study

**DOI:** 10.3389/fpubh.2022.866107

**Published:** 2022-04-08

**Authors:** Jean Claude Katte, Fanny Morfaw-Kibula, Batakeh B. Agoons, Sylvain Zemsi, Magellan Guewo-Fokeng, Eugene Sobngwi

**Affiliations:** ^1^National Obesity Centre and Endocrinology and Metabolic Diseases Unit, Yaounde Central Hospital, Yaounde, Cameroon; ^2^Department of Programme and Training, RSD Institute, Yaoundé, Cameroon; ^3^Radiology Unit, Logbaba District Hospital, Douala, Cameroon; ^4^Internal Medicine Unit, Bafang District Hospital, Bafang, Cameroon; ^5^Department of Biochemistry, Faculty of Medicine and Biomedical Sciences, University of Yaounde 1, Yaounde, Cameroon; ^6^Department of Internal Medicine and Specialities, Faculty of Medicine and Biomedical Sciences, University of Yaounde 1, Yaounde, Cameroon

**Keywords:** Urinary C-peptide to creatinine ratio, type 1 diabetes, type 2 diabetes, ketosis-prone diabetes, sub-Saharan Africa

## Abstract

**Background:**

The clinical utility of Urinary C-Peptide to Creatinine Ratio (UCPCR) is well understood in people with different types of diabetes in Caucasian populations, but studies are lacking in African populations. We, therefore, aimed to examine Urinary C-Peptide to Creatinine Ratio levels among groups of people with different types of diabetes in a sub-Saharan African population.

**Methods:**

A total of 47 adults with diabetes; 10 with type 1 diabetes, 26 with type 2 diabetes, 11 with ketosis-prone diabetes, and 22 healthy control individuals, were recruited from Yaoundé Central Hospital in Cameroon. Fasting blood glucose and C-peptide were measured in venous blood and urine. Stimulated Urinary C-Peptide to Creatinine Ratio was determined in all subjects after ingestion of a standardized mixed meal. We compared the stimulated Urinary C-peptide to Creatinine Ration concentration in subjects with type 1 diabetes to the other groups.

**Results:**

The basal C-peptide and HOMA-β were lower in T1D than in the T2D group [median 57 (34, 69) vs. 398 (335, 502) pmol/l; *p* ≤ 0.001] and [median 3.0 (1.63, 5.25) vs. 30.6 (17.94, 45.03); *p* < 0.001] respectively. Also, basal C-peptide and HOMA-β were lower in T1D than in those with KPD [median 57 (34, 69) vs. 330 (265, 478) pmol/l; *p* = 0.003] and [median 3.0 (1.63, 5.25) vs. 47.1 (16.2, 63.1), *p* = 0.001] respectively. Basal C-peptide was not different between participants with T2D and KPD; 398 (335, 502) vs. 330 (265, 478) pmol/l, *p* = 0.19. Stimulated UCPCR was lower in T1D compared to T2D, KPD and control participants; [median 0.29 (0.14, 0.68) vs. 0.89 (0.40, 1.69) nmol/moll; *p* = 0.009], [median 0.29 (0.14, 0.68) vs. 1.33 (0.84, 1.59) nmol/mol; *p* = 0.006] and [median 0.29 (0.14, 0.68) vs. 1.21 (0.85, 1.21) nmol/mol; *p* = 0.005] respectively. However, stimulated UCPCR was similar between the T2D and KPD study participants; 0.89 (0.40, 1.69) vs. 1.33 (0.84, 1.59) nmol/mol, *p* = 0.36.

**Conclusions:**

Stimulated Urinary C-Peptide to Creatinine Ratio (UCPCR) is lower in participants with type 1 diabetes compared to those with other types of diabetes in this population. This means stimulated UCPCR could potentially differentiate type 1 diabetes from other diabetes types among people with diabetes in sub-Saharan Africa.

## Introduction

### Background

Differentiating between different diabetes types is usually challenging in sub-Saharan Africa, where clinicians rely solely on clinical judgment (1). Moreover, apart from type 1 and type 2 diabetes, other atypical forms of diabetes, such as ketosis-prone diabetes (KPD), are prevalent in the region (2). While type 1 diabetes is exclusively treated with the exogenous administration of insulin, people with type 2 diabetes and KPD can be treated with oral anti-diabetic tablets. They may, however, need insulin for optimal glycaemic control and in other specific circumstances (3). Therefore, differentiating between the different types of diabetes is crucial for deciding therapeutic strategies for optimal management, and the measurement of endogenous insulin secretion has been shown to be helpful (4, 5).

C-peptide, secreted in equal amounts with insulin from the pancreas, is often used in clinical settings to measure endogenous insulin secretion (5). Its measurement in the blood (serum C-peptide) is considered the reference method (during a mixed meal tolerance test) but is cumbersome and expensive to perform (6). Urine C-peptide measurement is a less invasive and practical alternative to serum C-peptide measurements (5). Furthermore, Urinary C-peptide to creatinine ratio (UCPCR) is a surrogate marker for endogenous insulin secretion (7). While the importance of UCPCR in diabetes classification and management has been well demonstrated in people with diabetes of European descent (8–10), very little or no work exists on the its clinical utility in people with diabetes in sub-Saharan Africa. We, therefore, carried out a preliminary analysis into examining UCPCR levels amongst people with different types of diabetes and healthy controls in a clinical setting in sub-Saharan Africa.

## Methods

### Study Setting, Participants, and Ethical Considerations

Forty-seven (47) adults with diabetes [10 with type 1 diabetes (T1D), 26 with type 2 diabetes (T2D), and 11 with ketosis-prone diabetes] and 22 healthy control participants were enrolled in the study. All participants provided written informed consent before the start of the study. T1D was defined as young-onset diabetes (age of onset <30 years) with the presence of glutamic acid decarboxylase autoantibody and on insulin treatment. T2D was defined as previously diagnosed diabetes managed by lifestyle measures and/or oral anti-diabetic tablets. Ketosis-prone diabetes (KPD) was defined as diabetes diagnosed in the state of significant ketosis (urine ketones ≥ 13.7 mmol/l), initially requiring insulin therapy to achieve optimal glucose control with absent glutamic acid decarboxylase autoantibody (11). All healthy control individuals had normal fasting blood glucose levels with no personal or family history of diabetes. The National Ethics Committee of Cameroon's Ministry of Public Health approved the study (N. 120/CNE/SE/09).

### Procedure

Participants were required to take their medications (including insulin) as prescribed by their clinicians the day before the test. However, they were advised to eat and take their last medication before 10 pm so that they should have well fasted upon arrival for clinical investigations. Upon arrival for clinical investigation, we verified that the participants had fasted for at least 8 h and immediately performed capillary fasting glucose. When the capillary fasting glucose was >14 mmol/l, the participant's treatment was adjusted with the help of his/her clinician, and another appointment was arranged. After a short interview, we measured and recorded anthropometric data (weight, height, BMI, body fat mass, percent fat mass, waist and hip circumferences) onto a pre-structured data collection form. Afterward, we collected fasting venous blood and urine samples from each participant for fasting blood glucose and basal C-peptide determination. Each participant later ingested a standard meal with an energetic value comparable to that of a boost to stimulate insulin secretion (12). Postprandial urine was collected 2 h after ingesting the standard meal to determine C-peptide and creatinine concentrations.

The participants did not take any medication on the day of the investigations. After completing the standard meal to determine the stimulated UCPCR, we performed a capillary glucose measurement and adjusted the dose of medication for the participants on insulin to cater for any high glucose levels. Participants on oral anti-diabetic drugs continued their drugs as prescribed by their clinicians.

### Anthropometric Measurements

Height was measured to nearest 0.5 cm, and weight in light clothes to the nearest 0.1 kg, and body mass index (BMI) was calculated as a ratio of weight in kg and height in meter-squared. Waist and hip circumference were measured to the nearest 0.5 cm. Total fat mass was measured by bio-impedancemetry (TANITA BC 420 MA, TANITA Corporation 1-14-2 Maeno-cho, Tabashi-ku, Tokyo-Japan).

### Biochemical Measurements

Plasma glucose was measured by the hexokinase method (Roche Diagnostics GmbH, Mannheim, Germany). HbA1c was measured using the validated HLC-723G7 automatic HbA1c analyzer (Japan Tosoh Corporation). Plasma and urine C-peptide levels were measured by an immunoradiometric assay (IRMA-C-PEP, CIS International) with an intra-assay CV of 3.7–6.6% and an interassay CV of 4.4–8.0%. Urine creatinine was measured using the Kinetic Jaffe method.

### Calculations

Urinary C-peptide to creatinine ratio (UCPCR) was calculated as a surrogate measurement of insulin secretion by dividing urine C-peptide and urine creatinine levels. Insulin secretory capacity was calculated as the HOMA-β cell index according to the equation: HOMA-β(C-P) = 0.27 x fasting C-peptide (nmol/l)/ [fasting glucose (mmol/l)-3.5] for people with diabetes; and HOMA- β (C-P) = 0.27 x basal C-peptide / (FPG-3.5 mmol) + 50 for the healthy control participants (13).

### Statistical Methods

Data were coded, entered, and analyzed using StataSE15. Results are presented as counts (percentages) and medians (interquartile range). Mann-Whitney U test was used to compare data between any two study groups. A *p*-value < 0.05 was set as statistically significant.

## Results

### Clinical and Biochemical Characteristics of the Study Population

A total of 69 participants were included in this study, 10 with T1D, 26 with T2D, 11 with KPD, and 22 healthy controls. Females and males were similar in proportion. Participants with type 1 diabetes were the youngest with a median age of 20 (18, 22) and duration of diabetes at 1.6 (1.2, 2.1) years. Table 1 shows the clinical and metabolic characteristics across the study groups.

**Table 1:** General characteristics of the study population.

	**T1D (*n* = 10)**	**T2D (*n* = 26)**	**KPD (*n* = 11)**	**Controls (*n* = 22)**
**Clinical characteristics**
Sex (M/F)	7/3	11/15	8/3	8/14
Age (years)	20 (18, 22)	55 (41, 61)	30 (25, 39)	32 (24, 53)
Diabetes duration (years)	1.6 (1.2, 2.1)	1.6 (0.9, 2.5)	4.1 (2.2, 9.8)	NA
Treatment, n				
Diet only	0	5 (19.2)	0	NA
OAD only	0	8 (30.8)	7 (63.6)	NA
Insulin only	10 (100)	8 (30.8)	1 (9.1)	NA
OAD + insulin	0	5 (19.2)	3 (27.3)	NA
BMI (kg/m^2^)	22.7 (20.5, 24.5)	30.2 (25.4, 31.3)	27.3 (24.6, 31.5)	25.4 (22.9, 27.2)
Fat mass (kg)	10.1 (4.8, 14.8)	26.9 (16.4, 30.7)	19.3 (10.4, 25.2)	18.5 (14.5, 25.9)
Fat (%)	13.5 (8.5, 21)	32.6 (25.1, 39.4)	22.8 (14.9, 27.5)	23.0 (20.0, 35.0)
Waist circumference (cm)	79 (78, 79)	97 (91, 105)	93 (84, 99)	83 (76, 94)
Hip circumference (cm)	92 (87, 96)	109 (98, 111)	101 (97, 111)	98 (92, 109)
**Metabolic characteristics**
FPG (mmol/l)	11.8 (7.3, 13.1)	7.1 (6.3, 8.5)	5.7 (4.8, 7.9)	5.2 (4.8, 5.4)
HOMA-β	3.0 (1.6, 5.2)	30.6 (17.9, 45.0)	47.1 (16.2, 63.1)	107.8 (93.4, 128.7)
HbA1c (%)	14.0 (10.9, 14.0)	7.8 (6.6, 9.5)	7.1 (5.8, 12.8)	5.8 (5.7, 6.0)
Basal C-peptide (pmol/l)	57 (34, 69)	398 (335, 502)	330 (265, 478)	364 (287, 432)
Basal UCPCR (nmol/mmol)	0.16 (0.04, 0.26)	0.43 (0.20, 0.60)	0.31 (0.16, 0.59)	0.39 (0.23, 0.67)
Stimulated UCPCR (nmol/mmol)	0.29 (0.14, 0.68)	0.89 (0.40, 1.69)	1.33 (0.84, 1.59)	1.21 (0.85, 1.21)

*T1D, type 1 diabetes; T2D, type 2 diabetes; KPD, Ketosis prone diabetes; M, male; F, female; BMI, Body Mass Index; FPG, fasting plasma glucose; HOMA-β, Homeostasis Model Assessment-Beta-cell function; HbA1C glycosylated hemoglobin; BCP, Basal C-peptide; OAD, Oral anti-diabetic drugs; UCPCR, Urinary C-peptide creatinine ratio. NA, Not applicable*.

*Data are in counts (percentage) and median (IQR)*.

### Comparison of Basal C-Peptide Among the Four Groups of Participants

**Figure 1:**
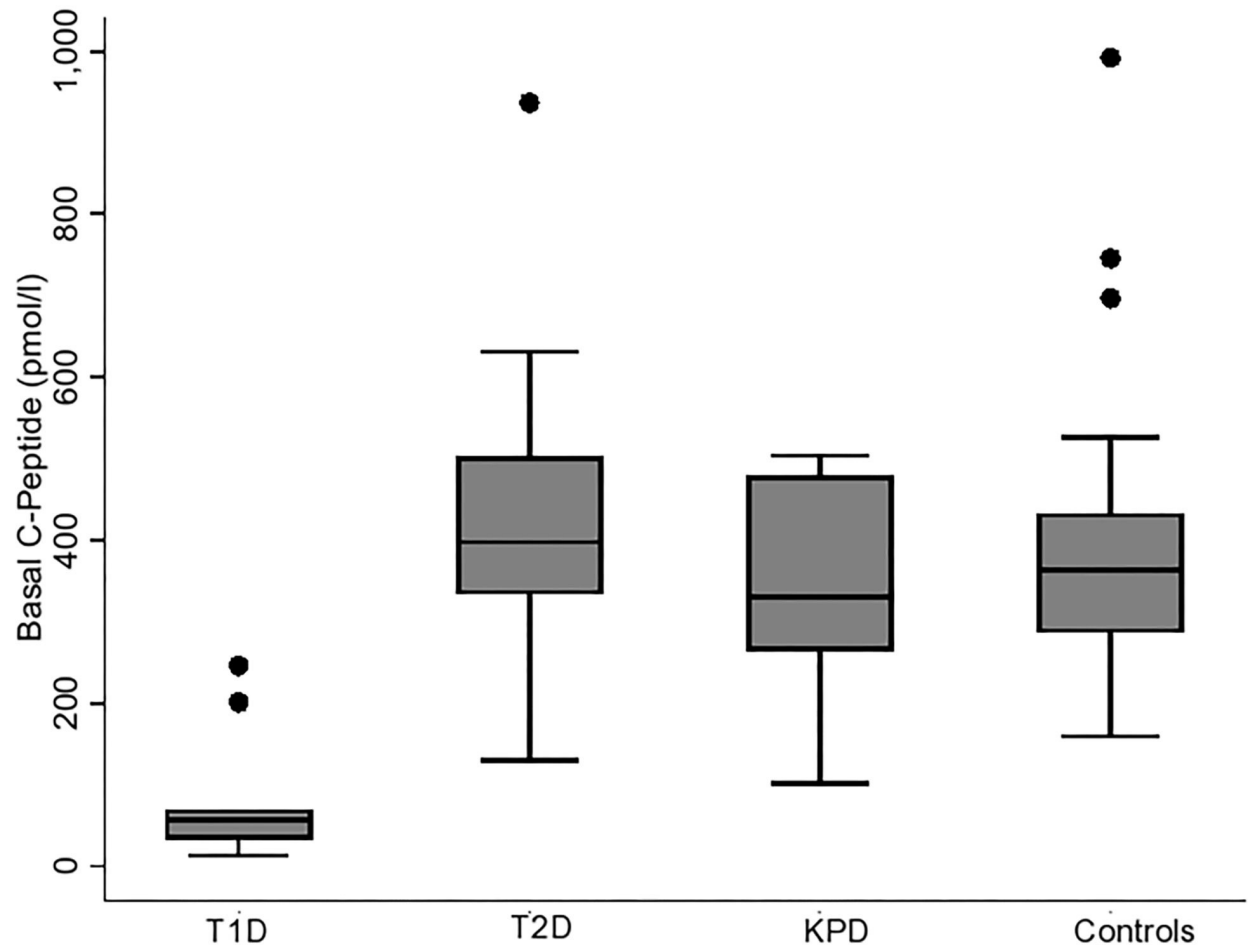
Boxplot showing the basal C-peptide levels in the different categories of participants. The error bars represent the standard errors.

**Figure 2:**
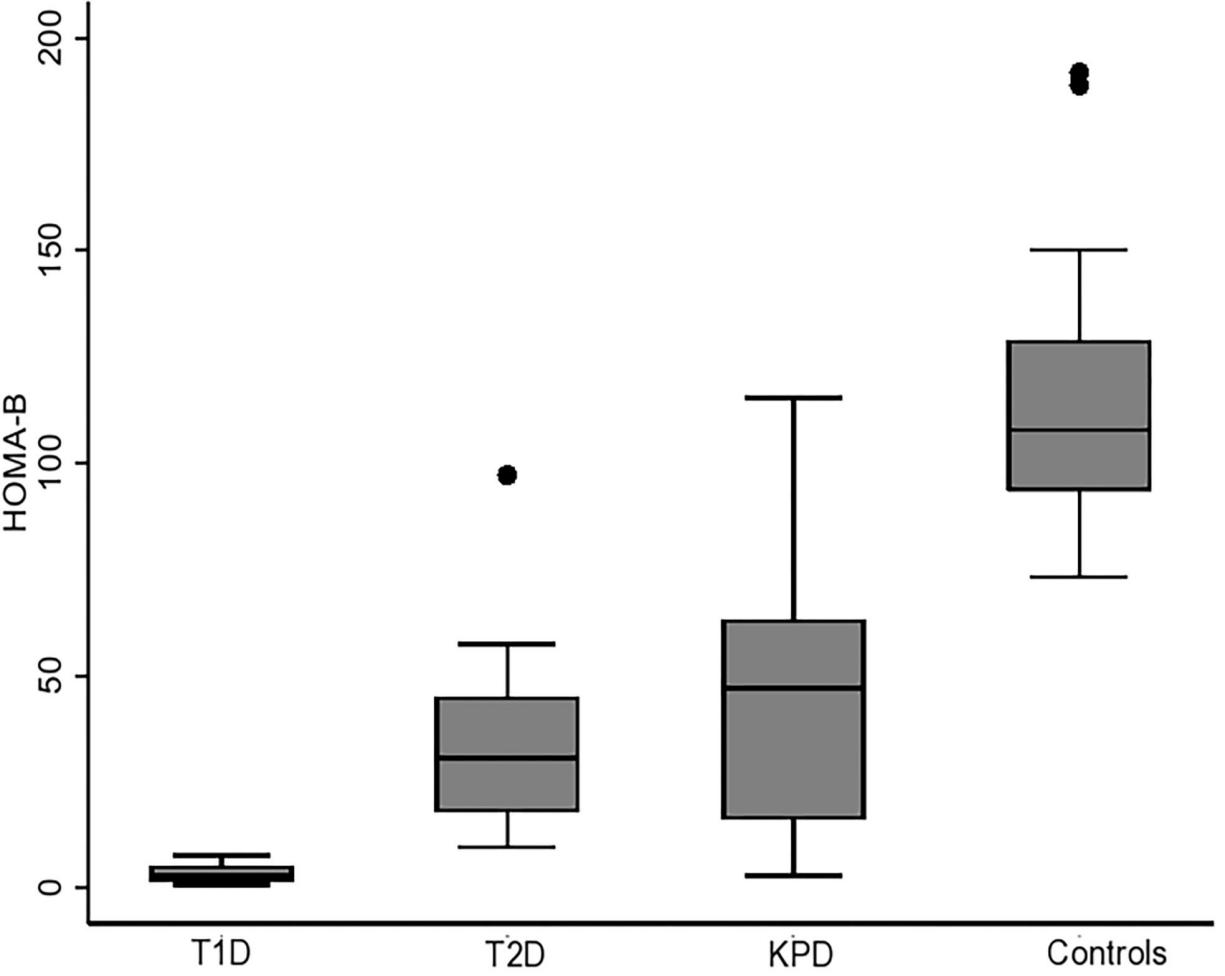
Boxplot showing HOMA-β (beta cell function) in the different categories of participants. The error bars represent the standard errors.

Figures 1, 2 show the basal C-peptide and HOMA-β levels between the different study groups. The basal C-peptide and HOMA-β were lower in T1D than in the T2D group [median 57 (34, 69) vs. 398 (335, 502) pmol/l; *p* ≤ 0.001] and [median 3.0 (1.63, 5.25) vs. 30.6 (17.94, 45.03); *p* < 0.001] respectively. Basal C-peptide and HOMA-β were lower in T1D than in those with KPD [median 57 (34, 69) vs. 330 (265, 478) pmol/l; *p* = 0.003] and [median 3.0 (1.63, 5.25) vs. 47.1 (16.2, 63.1), *p* = 0.001] respectively. Basal C-peptide and HOMA-β were lower in T1D than in controls [median 57 (34, 69) vs. 364 (287, 432) pmol/l; *p* < 0.001] and [median 3.0 (1.63, 5.25) vs. 107.8 (93.4, 128.7), *p* < 0.001] respectively. Basal C-peptide was not significantly different between participants with T2D and KPD; 398 (335, 502) vs. 330 (265, 478) pmol/l, *p* = 0.19.

### Comparison of Basal and Stimulated UCPCR Among the Four Groups of Participants

**Figure 3:**
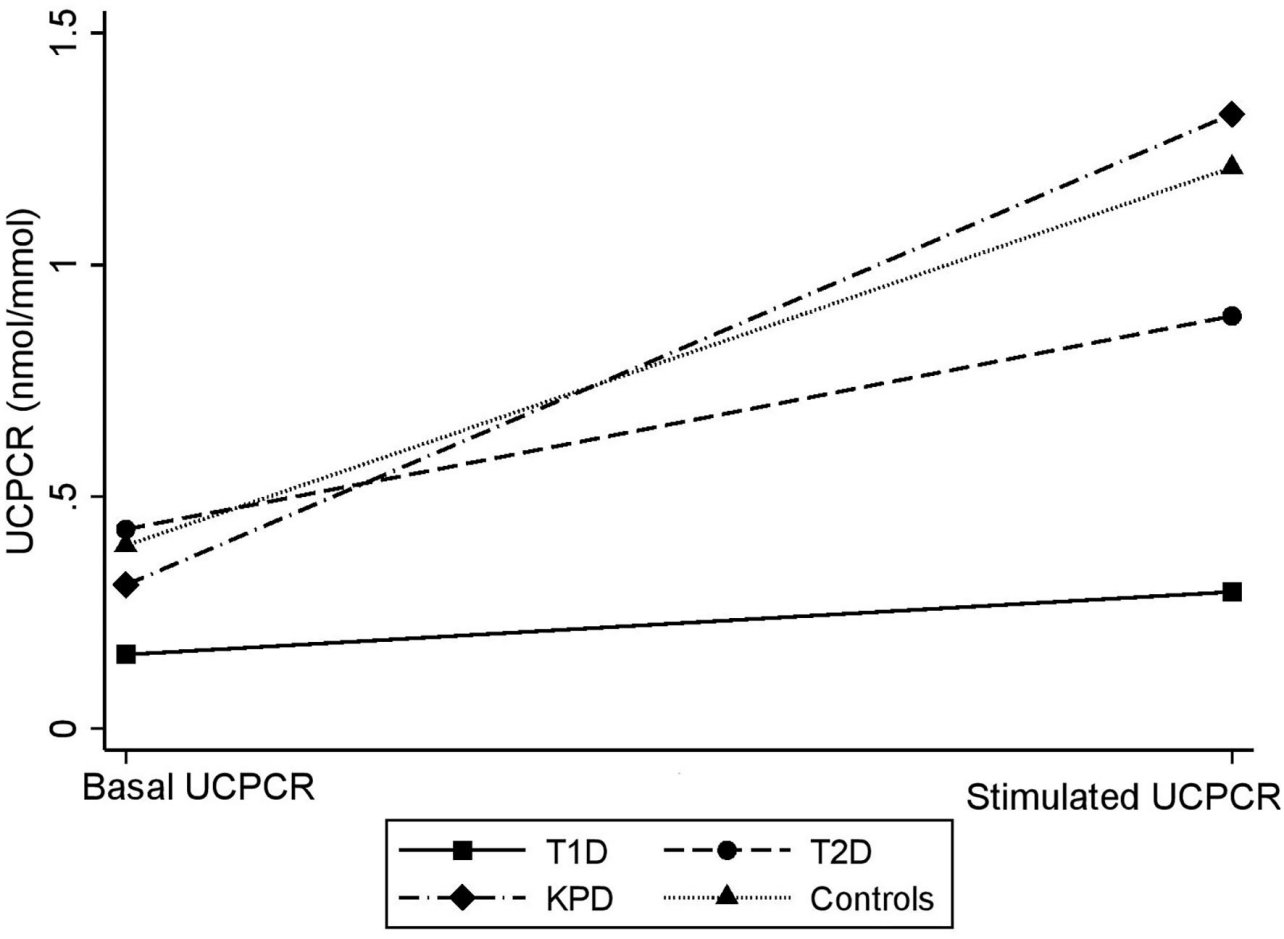
Scatter showing difference in basal and stimulated UCPCR values across the different study groups. The gradient is lowest in participants with T1D.

**Figure 4:**
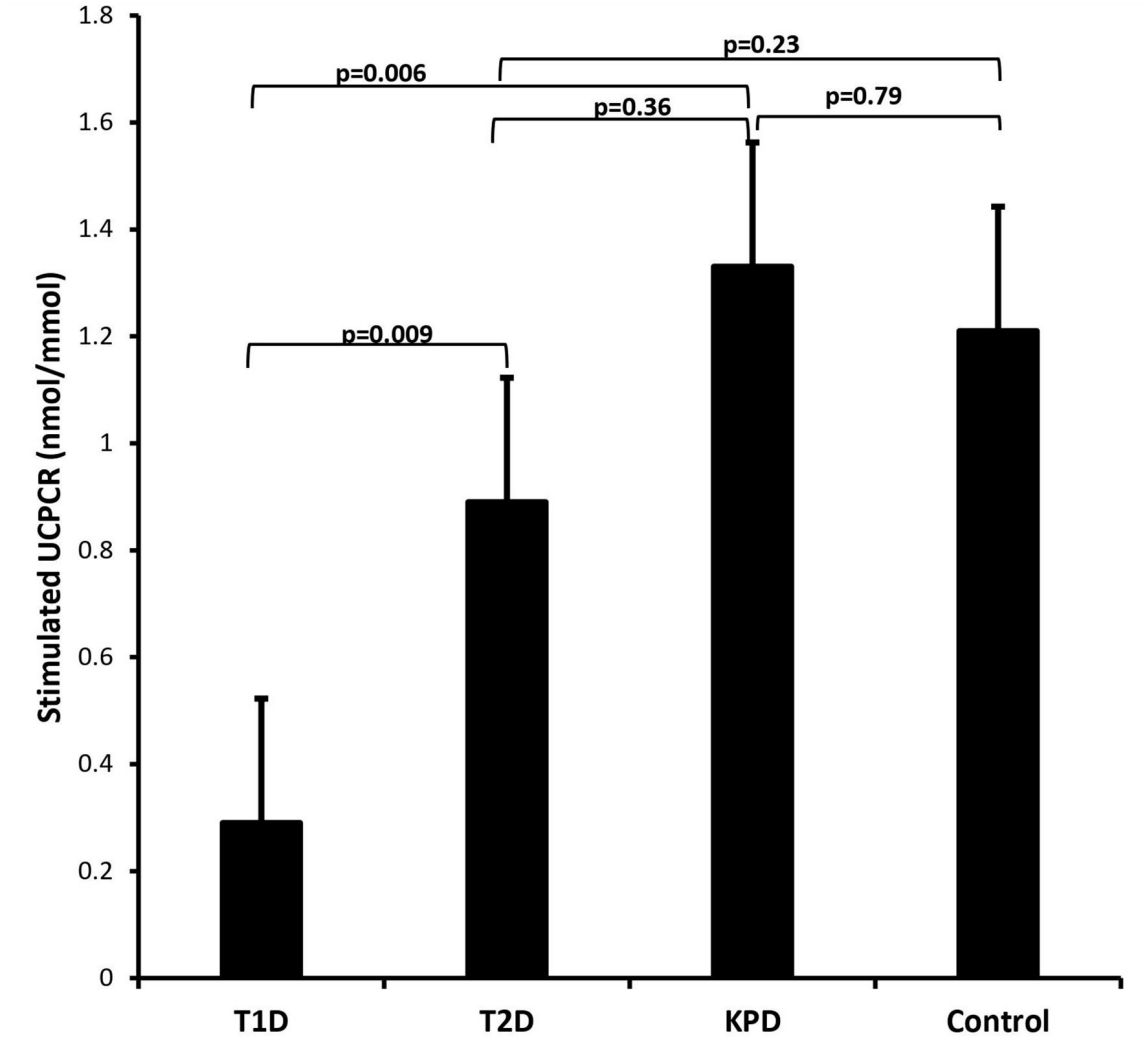
Barchart showing that stimulated UCPCR is lowest in participants with type 1 diabetes compared to those with type 2 and ketosis-prone diabetes. The error bars represent the standard errors.

Figure 3 is a scatter plot showing that stimulated UCPCR is lowest in participants with type 1 diabetes compared to those with type 2 diabetes and ketosis-prone diabetes. Figure 4 shows the comparison of the stimulated UCPCR between two different different study groups. Stimulated UCPCR was lower in T1D compared to T2D, KPD and control participants; [median 0.29 (0.14, 0.68) vs. 0.89 (0.40, 1.69) nmol/moll; *p* = 0.009], [median 0.29 (0.14, 0.68) vs. 1.33 (0.84, 1.59) nmol/mol; *p* = 0.006] and [median 0.29 (0.14, 0.68) vs. 1.21 (0.85, 1.21) nmol/mol; *p* = 0.005] respectively. Stimulated UCPCR was similar between the T2D and KPD study participants; 0.89 (0.40, 1.69) vs. 1.33 (0.84, 1.59) nmol/mol, *p* = 0.36.

## Discussion

This study aimed to examine UCPCR levels amongst groups of people with different types of diabetes in a sub-Saharan African population. We found that stimulated UCPCR after a standard meal test was lower in people with type 1 diabetes compared to those with type 2 diabetes and ketosis-prone diabetes. UCPCR is a simple and less invasive method of assessing endogenous insulin secretion and may be potentially relevant in identifying different types of diabetes in resource-limiting settings where complex laboratory methods are scarce or expensive.

This study has several limitations. First, the small sample size within the individual groups limits the ability to make firm conclusions about the similarities or differences observed between groups. Secondly, those identified as having KPD may just be patients with type 2 diabetes with a younger age of onset since it is known that type 2 diabetes patients can manifest ketosis. Thirdly, we did not use boric acid urine containers that offer 72-h stability to urine C-peptide (14), and therefore the observed UCPCR values may have been underestimated due to stability issues. Also, due to the limited sample size, we could not carry out discriminative analysis with sensitivity and specificity calculations for UCPCR in defining absolute severe insulin deficiency, which is the hallmark for type 1 diabetes. However, this is one of the first studies to the best of our knowledge to examine the clinical relevance of UCPCR different groups of people with diabetes in sub-Saharan Africa.

Several authors have shown that a basal plasma C-peptide cut-off of 0.08 nmol/l (and/or stimulated UCPCR cut-off of 0.2 nmol/mol) is a reliable threshold of clinical importance in diabetes classification and management. It has also been suggested that these cut-offs can also be clinically relevant in African settings (5, 15). Generally, a stimulated C-peptide of <0.2 nmol/l in plasma or 0.2 nmol/mol in urine in people with diabetes indicates severe insulin deficiency and, therefore, permanent insulin requirement (16).

The type 1 diabetes participants in our study had substantially higher baseline glucose and HbA1c values than the other participants. Achieving glycaemic control is a daunting task for individuals with type 1 diabetes worldwide, but more so for those living in resource-limited settings where about (17, 18). Chronic hyperglycemia (glucose toxicity) typically negatively affects beta-cell function, leading to poor insulin secretion, a state commonly referred to as beta-cell exhaustion or dysfunction (19). However, in type 1 diabetes, it may be challenging to ascertain whether the level of insulin secretion observed is affected by glucose toxicity or just a function of the remaining functional beta cells. However, to limit the possibility that glucose toxicity should impact residual insulin secretion, we performed the meal test only in participants with a fasting glucose level of <14 mmol/l on the morning of the test.

Stimulated UCPCR has previously been shown to differentiate between type 1 and other forms of diabetes in Caucasian populations (8, 10, 20). Besser et al. showed that post-home meal UCPCR could discriminate type 1 from type 2 diabetes and MODY, a specific form of atypical diabetes presentation (20). The prevalence of MODY in sub-Saharan Africa is unknown but may be lower than that of ketosis-prone diabetes. The study also showed that UCPCR could not discriminate between type 2 diabetes and MODY (20). Our study found that stimulated UCPCR levels were similar between type 2 diabetes and KPD. This finding underscores a major overlap between these two clinical entities when insulin secretion is taken into consideration (21). In this study, both T2D and KPD participants had similar clinical features such as BMI, waist, and hip circumferences, although KPD participants were younger than those with type 2 diabetes.

Ketosis-prone diabetes, at times called “Flatbush diabetes” is highly prevalent in sub-Saharan Africa and populations of African descent (22, 23). People with KPD have clinical features of type 1 and type 2 diabetes and have sporadic periods of insulin requirement marked by insulin deficiency, which may lead to severe ketosis if not appropriately treated (24). Participants identified as having KPD in this study were probably in remission, which could be evidenced by their HOMA-B and stimulated UCPCR values being similar to that seen in the type 2 diabetes group. KPD patients will usually present with severe ketosis, high insulin requirements, and lowered basal C-peptide and insulin secretory capacity during a ketotic crisis than their non-ketotic counterparts (25). Generally, as in type 2 diabetes patients, KPD patients have been demonstrated to have reduced insulin secretory capacity compared to healthy matched controls even during remission (21).

In summary, stimulated UCPCR levels were lower in participants with type 1 compared to those with type 2 diabetes and ketosis-prone diabetes. Stimulated UCPCR levels were similar amongst participants with type 2 diabetes and ketosis-prone diabetes. However, a large sample size and formal mixed meal tolerance test studies are needed in sub-Saharan African populations to robustly define the usefulness of UCPCR in this setting with prevalent atypical diabetes types.

## Data Availability Statement

The raw data supporting the conclusions of this article is readily available upon reasonable request from the corresponding author.

## Ethics Statement

This study involving human participants was reviewed and approved by the National Ethics Committee of Cameroon's Ministry of Public Health (No 120/CNE/SE/09). All participants provided a written signed informed consent prior to participating in the study.

## Author Contributions

JCK, SZ, MG-F, and ES designed the study. BA, FM-K, SZ, and MG-F collected data. JCK, FM-K, and MG-F analyzed the data. BA, JCK, SZ, and ES built the manuscript. The study was done under the supervision of ES. All authors revised the manuscript and read and approved the final manuscript.

## Conflict of Interest

The authors declare that the research was conducted in the absence of any commercial or financial relationships that could be construed as a potential conflict of interest.

## Publisher's Note

All claims expressed in this article are solely those of the authors and do not necessarily represent those of their affiliated organizations, or those of the publisher, the editors and the reviewers. Any product that may be evaluated in this article, or claim that may be made by its manufacturer, is not guaranteed or endorsed by the publisher.

## References

[1] Mbanya JC Motala AA Sobngwi E Assah FK Enoru ST. Diabetes in sub-Saharan Africa. Lancet. (2010) 375:2254–66. 10.1016/S0140-6736(10)60550-8 20609971

[2] Sobngwi E Mauvais-Jarvis F Vexiau P Mbanya JC Gautier JF. Diabetes in Africans. Part 2: ketosis-prone atypical diabetes mellitus. Diabetes Metab. (2002) 28:5–12. 11938022

[3] Atun R Davies JI Gale EAM Barnighausen T Beran D Kengne AP. Diabetes in sub-Saharan Africa: from clinical care to health policy. Lancet Diabetes Endocrinol. (2017) 5:622–67. 10.1016/S2213-8587(17)30181-X 28688818

[4] Shields BM Peters JL Cooper C Lowe J Knight BA Powell RJ. Can clinical features be used to differentiate type 1 from type 2 diabetes? A systematic review of the literature. BMJ Open. (2015) 5:e009088. 10.1136/bmjopen-2015-009088 26525723 PMC4636628

[5] Jones AG Hattersley AT. The clinical utility of C-peptide measurement in the care of patients with diabetes. Diabet Med. (2013) 30:803–17. 10.1111/dme.12159 23413806 PMC3748788

[6] Leighton E Sainsbury CA Jones GC. A Practical review of C-peptide testing in diabetes. Diabetes Ther. (2017) 8:475–87. 10.1007/s13300-017-0265-4 28484968 PMC5446389

[7] McDonald TJ Perry MH. Detection of C-peptide in urine as a measure of ongoing beta cell function. Methods Mol Biol. (2016) 1433:93–102. 10.1007/7651_2016_330 27083170

[8] Yilmaz Agladioglu S Sagsak E Aycan Z. Urinary C-peptide/creatinine ratio can distinguish maturity-onset diabetes of the young from type 1 diabetes in children and adolescents: a single-center experience. Horm Res Paediatr. (2015) 84:54–61. 10.1159/000375410 25792383

[9] Hope SV Knight BA Shields BM Hattersley AT McDonald TJ Jones AG. Random non-fasting C-peptide: bringing robust assessment of endogenous insulin secretion to the clinic. Diabet Med. (2016) 33:1554–8. 10.1111/dme.13142 27100275 PMC5226330

[10] Elzahar W Arafa A Youssef A Erfan A El Amrousy D. Urinary C-peptide creatinine ratio to differentiate type 2 diabetes mellitus from type 1 in pediatric patients. Eur J Pediatr. (2020) 179:1115–20. 10.1007/s00431-020-03606-7 32052124

[11] Mauvais-Jarvis F Sobngwi E Porcher R Riveline JP Kevorkian JP Vaisse C. Ketosis-prone type 2 diabetes in patients of sub-Saharan African origin: clinical pathophysiology and natural history of beta-cell dysfunction and insulin resistance. Diabetes. (2004) 53:645–53. 10.2337/diabetes.53.3.645 14988248

[12] Jean-Claude Katte Virginie Poka-Mayap Anxious Niwaha Wisdom Nakanga Angus Jones Timothy J. McDonald. Post-meal urinary C-peptide creatinine ratio is a moderate measure of insulin secretion in diabetes patients in Cameroon: results from a cross-sectional study. PAMJ Clinical Medicine. (2020) 3:12. 10.11604/pamj-cm.2020.3.12.2241926401219

[13] Li X Zhou ZG Qi HY Chen XY Huang G. [Replacement of insulin by fasting C-peptide in modified homeostasis model assessment to evaluate insulin resistance and islet beta cell function]. Zhong Nan Da Xue Xue Bao Yi Xue Ban. (2004) 29:419–23. 16134594

[14] McDonald TJ Knight BA Shields BM Bowman P Salzmann MB Hattersley AT. Stability and reproducibility of a single-sample urinary C-peptide/creatinine ratio and its correlation with 24-h urinary C-peptide. Clin Chem. (2009) 55:2035–9. 10.1373/clinchem.2009.129312 19713273

[15] Siraj ES Reddy SS Scherbaum WA Abdulkadir J Hammel JP Faiman C. Basal and postglucagon C-peptide levels in Ethiopians with diabetes. Diabetes Care. (2002) 25:453–7. 10.2337/diacare.25.3.453 11874929

[16] Steffes MW Sibley S Jackson M Thomas W. Beta-cell function and the development of diabetes-related complications in the diabetes control and complications trial. Diabetes Care. (2003) 26:832–6. 10.2337/diacare.26.3.832 12610045

[17] Bonora BM Boscari F Avogaro A Bruttomesso D Fadini GP. Glycaemic control among people with type 1 diabetes during lockdown for the SARS-CoV-2 outbreak in Italy. Diabetes Ther. (2020) 11:1369–79. 10.1007/s13300-020-00829-7 32395187 PMC7213551

[18] Djonou C Tankeu AT Dehayem MY Tcheutchoua DN Mbanya JC Sobngwi E. Glycemic control and correlates in a group of sub Saharan type 1 diabetes adolescents. BMC Res Notes. (2019) 12:50. 10.1186/s13104-019-4054-1 30670077 PMC6341641

[19] Cerf ME. Beta cell dysfunction and insulin resistance. Front Endocrinol. (2013) 4:37. 10.3389/fendo.2013.00037 23542897 PMC3608918

[20] Besser RE Shepherd MH McDonald TJ Shields BM Knight BA Ellard S. Urinary C-peptide creatinine ratio is a practical outpatient tool for identifying hepatocyte nuclear factor 1-{alpha}/hepatocyte nuclear factor 4-{alpha} maturity-onset diabetes of the young from long-duration type 1 diabetes. Diabetes Care. (2011) 34:286–91. 10.2337/dc10-1293 21270186 PMC3024335

[21] Choukem SP Sobngwi E Boudou P Fetita LS Porcher R Ibrahim F. beta- and alpha-cell dysfunctions in africans with ketosis-prone atypical diabetes during near-normoglycemic remission. Diabetes Care. (2013) 36:118–23. 10.2337/dc12-0798 22933436 PMC3526247

[22] Kitabchi AE. Ketosis-prone diabetes–a new subgroup of patients with atypical type 1 and type 2 diabetes? J Clin Endocrinol Metab. (2003) 88:5087–9. 10.1210/jc.2003-031656 14602730

[23] Banerji MA. Diabetes in African Americans: unique pathophysiologic features. Curr Diab Rep. (2004) 4:219–23. 10.1007/s11892-004-0027-3 15132889

[24] Sjoholm A. Ketosis-prone type 2 diabetes: a case series. Front Endocrinol (Lausanne). (2019) 10:684. 10.3389/fendo.2019.00684 31749761 PMC6843078

[25] Lontchi-Yimagou E Nguewa JL Assah F Noubiap JJ Boudou P Djahmeni E. Ketosis-prone atypical diabetes in Cameroonian people with hyperglycaemic crisis: frequency, clinical and metabolic phenotypes. Diabet Med. (2017) 34:426–31. 10.1111/dme.13264 27657549

